# Blood Pressure Morphology Assessment from Photoplethysmogram and Demographic Information Using Deep Learning with Attention Mechanism

**DOI:** 10.3390/s21062167

**Published:** 2021-03-19

**Authors:** Nicolas Aguirre, Edith Grall-Maës, Leandro J. Cymberknop, Ricardo L. Armentano

**Affiliations:** 1 GIBIO, Facultad Regional Buenos Aires, Universidad Tecnológica Nacional, Ciudad Autónoma Buenos Aires C1179AAQ, Argentina; ljcymber@frba.utn.edu.ar (L.J.C.); armen@frba.utn.edu.ar (R.L.A.); 2 LIST3N, Université de Technologie de Troyes, 10004 Troyes, France; edith.grall@utt.fr

**Keywords:** photoplethysmography, continuous arterial blood pressure, cuff-less calibration, deep learning

## Abstract

Arterial blood pressure (ABP) is an important vital sign from which it can be extracted valuable information about the subject’s health. After studying its morphology it is possible to diagnose cardiovascular diseases such as hypertension, so ABP routine control is recommended. The most common method of controlling ABP is the cuff-based method, from which it is obtained only the systolic and diastolic blood pressure (SBP and DBP, respectively). This paper proposes a cuff-free method to estimate the morphology of the average ABP pulse ($\bar{ABPM}$) through a deep learning model based on a seq2seq architecture with attention mechanism. It only needs raw photoplethysmogram signals (PPG) from the finger and includes the capacity to integrate both categorical and continuous demographic information (DI). The experiments were performed on more than 1100 subjects from the MIMIC database for which their corresponding age and gender were consulted. Without allowing the use of data from the same subjects to train and test, the mean absolute errors (MAE) were 6.57 ± 0.20 and 14.39 ± 0.42 mmHg for DBP and SBP, respectively. For $\bar{ABPM}$, R correlation coefficient and the MAE were 0.98 ± 0.001 and 8.89 ± 0.10 mmHg. In summary, this methodology is capable of transforming PPG into an ABP pulse, which obtains better results when DI of the subjects is used, potentially useful in times when wireless devices are becoming more popular.

## 1. Introduction

Cardiovascular diseases (CVDs) remain the most common cause of morbidity and mortality worldwide [1]. One of its main risk factors which reaches at least 1.3 billion people is high blood pressure (BP) or hypertension [2]. Unfortunately, most of the population is not aware of suffering a CVD until an event such as arrhythmia, heart attack, or stroke occurs. In this context, regular BP monitoring becomes an essential strategy of prevention, detection, and control for health.

Methods for measuring the BP are divided into noninvasive and invasive. The traditional noninvasive method involves the sphygmomanometry technique. In general, the measurement is carried out by a physician or different members of a clinical staff, and the subject to be measured rests for a few minutes in order to stabilize his BP. As it depends on an inflatable cuff, it does not serve as a continuous measurement method due to only two values are obtained: diastolic BP (DBP) and systolic BP (SBP). Invasive methods are performed by inserting intravascular catheters with pressure transducers. They have the disadvantage of exposing the subject to bleeding and infections. The advantage is the access to the continuous arterial BP (ABP) morphology, the gold standard for monitoring the BP. Additionally, a noninvasive practice to estimate the ABP is the tonometry technique in combination with the cuff sphygmomanometer. The tonometry technique provides the estimation of the waveform and the cuff sphygmomanometer provides calibrated values [3].

ABP morphology (ABPM) is defined by the mechanical interaction between the blood flow, originated in the hearth, and the arteries. The DBP is referenced to the minimum value of BP and it is related to the aortic valve opening to blood ejection. The SBP is defined as the maximum pressure value applied by the left ventricle in the heart’s cycle. It is the result of the interaction between the blood ejected into the arterial tree and the reflected waves [4]. The dicrotic notch (DN) represents the closure of the aortic valve and is used to calculate the duration of the ejection period and the beginning of the diastolic phase. ABPM can suffer of local alterations, such as those induced by the respiratory rhythm or specific vascular test maneuvers. On the other hand, permanent alterations can be observed as a result of advanced age or the appearance of vascular pathologies such as arterial stiffness [5]. In addition, the ABMP changes according to the site of the arterial tree at which it is measured. However, if both the waveform and calibration values are known, it is possible to use generalized transfer functions to estimate the ABPM at another site [6]. Furthermore, it is known that ABPM may be more predictive of cardiovascular events than just cuff-pressure values [7,8,9], may alert of CVDs such as diastolic dysfunction [10], or could be a valuable measure of the response to the treastmen of the obstructive sleep apnea [11]. In this sense, through the analysis of the ABPM it is possible to derive many features related to the health of the cardiovascular system [3]. Some of them correspond specifically to ABP values and other ones to temporal occurrences.

An important temporal feature introduced in [12] and studied more in depth in Mukkamala et al. [13] is the pulse transit time (PTT). It is defined as the time between the beginning of a pulse originated in the heart and its arrival at a specific point on the periphery of the artery tree. PTT shows a relationship with arterial pulse wave velocity (PWV) based on Moens–Korteweg equation:

(1) $$PWV=L/PTT=\sqrt{Eh/\rho 2r}$$

in which *E* is the elastic module, *h* is the arterial wall thickness, ρ is the blood density, and *r* is the radius of a vessel. And PWV can be related to ABP by Hughes equation [14]:

(2) $$E=E_{0}e^{\alpha P}$$

where both α > 0 and $E_{0}$ are subject-specific constants. $E_{0}$ corresponds to the zero-pressure modulus of the vessel wall and P reference to BP. PTT can also be defined as the difference between pulse arrival time (PAT) and the pre-ejection period (PEP). In this sense, PAT can be assessed as the time delay between the electrocardiogram’s (ECG) R-peak and the BP pulse onset. However, PAT is not expressed in Equation (1) and cannot be related directly to BP. Furthermore, it is shown that PEP represents a significant and variable proportion of PTT, from 10% to 30% [15]. Nevertheless, PAT is widely used by researchers as a good approximation of PTT, mainly due to the ease of its measurement [13].

Following this approach, in recent years there has been an increase in the amount of publications regarding the estimation of BP values in a noninvasive and real-time way, also called "cuff-less calibration". In this context, finger photoplethysmography (PPG) signal, due to its similarities in time and frequency domains [16] with BP, emerges as an interesting measurement for estimating BP. PPG is an optical device that measures the change of blood volume in the vessel. Its advantages are the low-cost, simplicity, and portability, very attractive characteristics for wearable devices [17]. Its disadvantages are the sensitivity to noise and artifact due to subject movements; therefore, a signal processing in general must be applied.

Unfortunately, PTT approach to estimate BP cannot be directly applied to PPG signal morphology. In order to deal with this issue, different approaches based on machine learning such as linear regression [18], AdaBoost [19], classical fully-connected neural network (NN) [20], and Gaussian process regression (GPR) [21] were proposed to modeling the subject-specific relation between PPG and BP. These techniques were focused in the feature extraction between PPG and ECG. In particular, in Monte-Moreno [22] and Ruiz-Rodríguez et al. [23] techniques called Random Forest (RF) and Deep Belief Network-Restricted Boltzmann Machine, respectively, were applied to estimate SBP and DBP extracting features from the PPG. On the contrary, with the disruption of deep learning techniques, feature extraction step could be relegated to the NN. In Eom et al. [24], raw ECG, PPG and ballistocardiogram (BCG) signals were used in a combined convolutional NN (CNN) and recurrent NN (RNN) model. Furthermore, some studies proposed to work only with PPG time-series [25] and its derivatives [26]. In Liang et al. [25], a pretrained CNN was used to classify three levels of hypertension based on the scalogram from the PPG and in Slapniča et al. [26], a spectro-temporal ResNet model was proposed to estimate the DBP and SBP values using the PPG in conjunction of the first and second derivatives (PPG and PPG, respectively). The latter was also a combination of CNN and RNN with gated recurrent units (GRU). Particularly, to the best of our knowledge, few studies aim to the hard task of directly estimate the continuous ABP. In Sideris et al. [27] and Sadrawi et al. [28] a RNN model, with long short-term memory (LSTM) units, and deep convolutional auto-encoder (DCAE) model, respectively, were proposed to transfer signals from PPG to ABP. General surveys on the existing and emerging approaches on this field can be found in Hosanee et al. [29] and Kyriacou [30].

In this context and considering the recommendations from Elgendi et al. [17], the collaborative spirit from Slapniča et al. [26] and the requirements when working with the MIMIC-III Matched Waveform Database (MWDB) and MIMIC-III Clinical Database (CDB) [31], the contributions of our work can be summarized in the next topics:

1. Morphology of the average ABP pulse ($\bar{ABPM}$): The proposed methodology has the capacity to estimate $\bar{ABPM}$, from which DBP, DN, and SBP values are then extracted.
2. Raw PPG signal and demographic information (DI): The proposed deep learning architecture allows the combination of the raw signal of the PPG and the DI age and gender of each subject in the same model. The addition of DI improves the estimation of $\bar{ABPM}$.
3. Limited bias: The quantities of records per subject and signals duration are limited to reduce subject’s biases.
4. Reproducibility: The processed dataset, subject’s ID, temporal information of each signal, model architecture, and training sources codes are available for reproducibility. Please see Supplementary Materials Section. The DI used due to requirements from [31] is not shared, but the codes to extract it if the request to access to MIMIC-III CDB is accepted, are also available.

## 2. Materials and Methods

**Figure 1:**
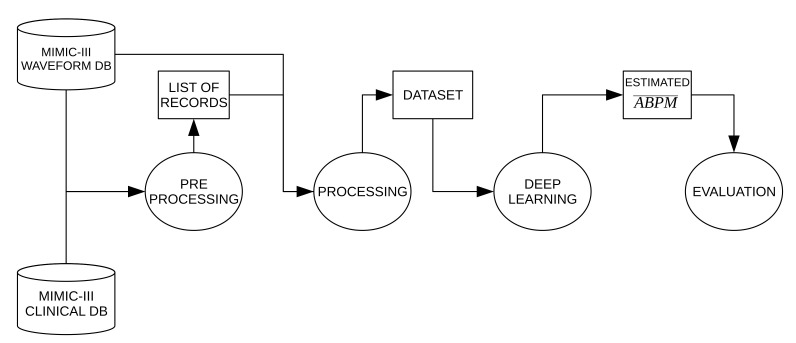
Block diagram of the proposed methodology.

Figure 1 shows a block diagram of the proposed methodology. Data for this work come from two public available databases: MIMIC-III MWDB and MIMIC-III CDB. The first one contains over 20,000 waveform records digitized at 125 Hz from more than 10,000 distinct patients in intensive care units and the second one includes information such as demographics, laboratory and microbiology test results, cardiology and radiology reports, and diagnostics. In preprocessing stage, records from MIMIC-III MWDB with invasive ABP and fingertip PPG signals are selected, then their corresponding subject’s age and gender were obtained from MIMIC-III CDB. In processing stage only segments with enough signal quality (SQ) are kept and each morphology of the average ABP pulse ($\bar{ABPM}$) is computed. Deep learning stage compromises the model architecture, hyperparameters settings, and training phase to get the estimated $\bar{ABPM}$ ($\hat{ABPM}$) from PPG signal. Finally, the values and time occurrences from $\hat{ABPM}$ are evaluated. Each stage will be detailed in the next subsections.

### 2.1. Preprocessing

The ID of the records with a minimum duration of 15 min and with both ABP and PPG signals were preserved. A 10 min interval was defined to consider the subject in a rest condition and 5 min was defined as a gap between different segments of the record. In this work only the age and gender were extracted from MIMIC-III CDB. The age of analysis was set between 18 and 89 years.

### 2.2. Processing

**Figure 2:**
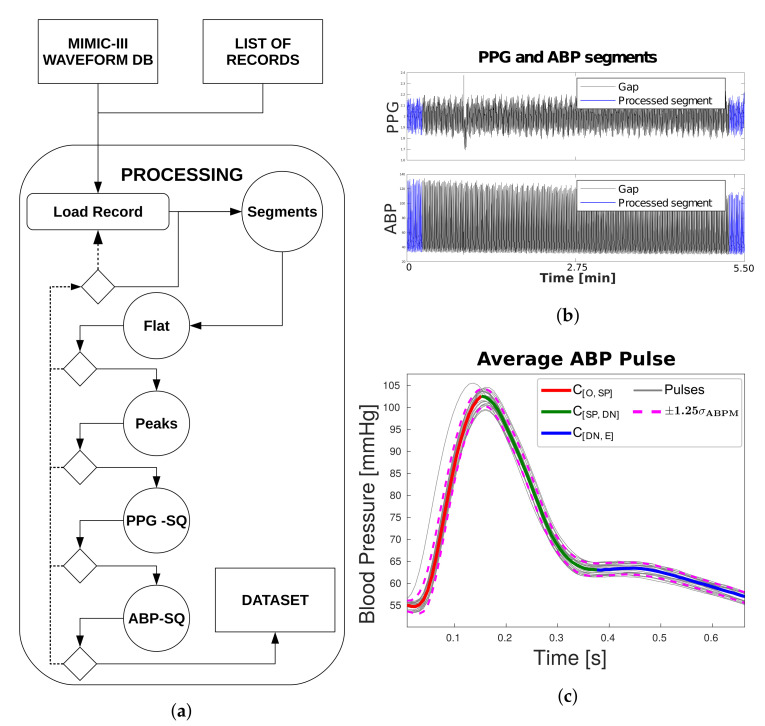
(**a**) Summarized processing stage. (**b**) In blue, two 15-s segments analyzed with 5 min gap between each other, in black. (**c**) An example of $\bar{ABPM}$ computed. Red, green, and blue lines correspond to the classes [onset—systolic peak], [systolic peak—dicrotic notch] and [dicrotic notch–end], respectively, while gray lines represent ABP pulses and dashed magenta lines represent the limits to consider the average pulse.

In Figure 2a is summarized the processing stage. Part of this section was inspired in the released code from Slapniča et al. [26]. Each record was loaded with the WFDB Toolbox [32] for Matlab and two 15-s segments spaced 5 min apart were analyzed (Figure 2b). Each time a segment was rejected for not meeting the requirements described below, a minute was waited before reanalyzing two new segments. If a record was not able to meet the criteria, it was excluded from the analysis and the next record was evaluated. If the criterion was met, a structured file containing both the raw segments and the processed pulses was generated.

The main steps were called *Flat*, *Peak*, *PPG-SQ*, and *ABP-SQ*. Several thresholds were set to ensure the quality for each segment and pulses, at least equal to those from Slapniča et al. [26]. Particularly, for *PPG-SQ* and *ABP-SQ* others were added. The pulses duration were limited in the range [0.5, 1.5] s considering normal physiological limits at rest. The number of pulses per segment analyzed was limited to [10, 30]. To be accepted, the difference SBP-DBP and the moment coefficient of skewness [33] had to be higher than 10 mmHg and zero, respectively.

More in detail, *Flat* and *Peak* detect null data and saturated points in valleys and peaks of signals respectively. Then, a Butterworth filter with cutoff frequencies [0.5, 8] Hz and MinMax normalization were performed only to the PPG segment. A pulse-by-pulse analysis was done with the marker proposed in Li et al. [34]. It is important to clarify that *PPG-SQ* corresponds to part of the feature extraction step in Slapniča et al. [26], but for this work it was only used for signal quality reasons. Once *PPG-SQ* was succeed, raw PPG segment was saved. The $\bar{ABPM}$ were calculated in *ABP-SQ* step. The ABP pulses were synchronized regarding their onsets. For each time-step t=iΔt, the mean ($\mu_{ABPM_{i}}$) and standard deviation ($\sigma_{ABPM_{i}}$) was calculated. Finally, $\bar{ABPM}$ was computed only with the points in range $\mu_{ABPM_{i}}\pm 1.25\sigma_{ABPM_{i}}$, as is shown in Figure 2c. Regarding the deep learning explained below, the class of each point related to different cardiac cycle stages was defined as one of the following intervals: [onset, systolic peak] ($C_{\left[O,SP\right]}$), [systolic peak, dicrotic notch ] ($C_{\left[SP,DN\right]}$) or [dicrotic notch, end] ($C_{\left[DN,E\right]}$).

**Figure 3:**
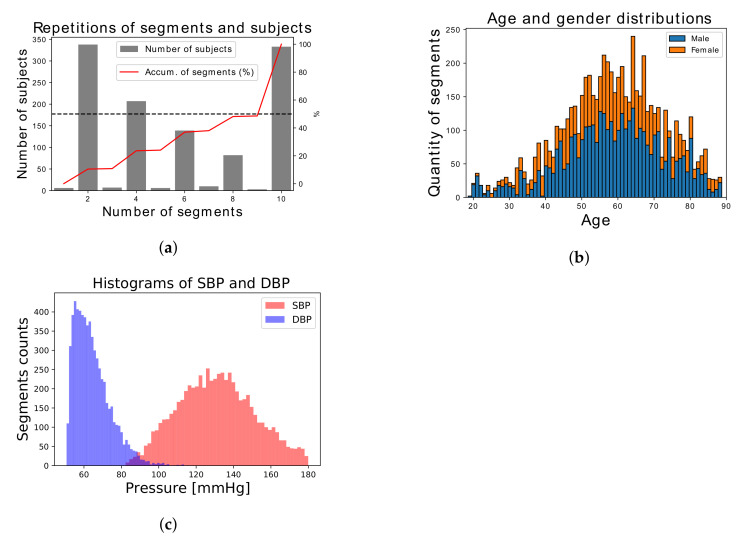
Selected dataset distributions: (**a**) Number of subjects with their corresponding quantity of segments (gray bars) and segments cumulative percentage (red line). (**b**) Age and gender. (**c**) Systolic blood pressure (SBP) and diastolic blood pressure (DBP).

Once all the records were processed, after a visual inspection, ABP and pulse duration were limited to 180 mmHg and 1.2 s, respectively. In addition, only pulses with skewness greater than 0.2 were accepted. At this point, there were 10,696 segments corresponding to 1131 subjects, where 169 subjects had more than 50% of segments. To reduce subject’s bias, the quantity of segments per subject was limited to 10. Finally, there were 6478 segments, where 333 subjects represent the 50% of segments (Figure 3a). Figure 3b,c show the age and gender distributions and DBP and SBP distributions, respectively, of the selected dataset. They were 464 females and 667 and males, while mean and standard deviation of age, DBP, and SBP were 58.6 ± 14.1 years, 64.48 ± 9.51 mmHg, and 130.84 ± 20.27 mmHg, respectively.

The raw PPG segment saved during processing was filtered before being used as input. A band-pass Butterworth filter, with cutoff frequencies [0.5, 45] Hz, was applied. As mentioned before, the PPG provides information that could improve $\hat{ABPM}$. PPG was computed using a Savitzky–Golay filter [35]. The window size and the polynomial degree was 7 and 3, respectively. In addition, one second was removed at the beginning and at the end of the segment to avoid artifacts caused by the two filters just mentioned. In summary, the dataset available for the deep learning stage was constituted by 6478 segments, 13 s each one, equivalent to 23.4 h.

### 2.3. Deep Learning

The proposed deep learning architecture is inspired by *seq2seq* encoder-decoder [36] models with attention mechanism [37,38] on the natural language processing domain. Before the detailed description of it in Section 2.3.3, a few concepts in relation with this model are described in Section 2.3.1. Furthermore, some considerations about the input data are presented in Section 2.3.2.

#### 2.3.1. RNN Encoder-Decoder

**Figure 4:**
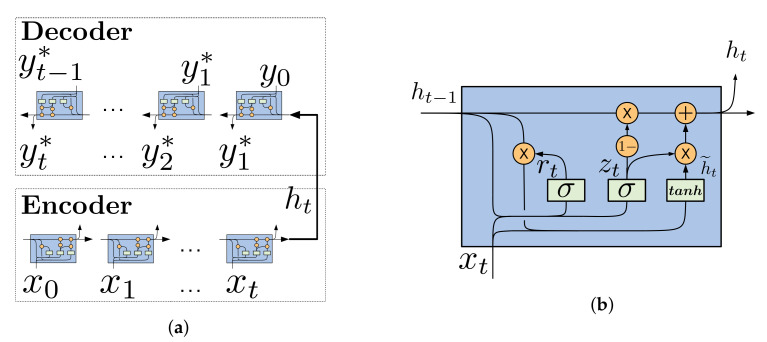
(**a**) recurrent NN (RNN) Encoder-Decoder architecture. (**b**) Structure of gated recurrent units (GRU) unit.

Encoder reads each input from a variable source sequence and encodes it into a fixed-length vector representation, also called hidden state. Then, the decoder starts initializing its own hidden state with the encoder one, and then generates at each time an output. Figure 4a shows an illustration of the encoder-decoder model using RNN, where the type of RNN selected for this work is called "gated recurrent unit" (GRU). The structure of GRU is shown in Figure 4b.

GRU structure was proposed by Cho et al. [36] to mitigate the vanishing/exploding gradient of the RNN. Input vectors of each GRU unit are the previous hidden state $h_{t-1}$ and the current input $x_{t}$, while the current hidden state $h_{t}$ correspond to the output. In this sense, $h_{t}$ is computed according to relations given by Equation (3):

(3) $$\begin{array}{cc} z_{t}= & \;\sigma \left(W_{z}\cdot \left[h_{t-1},\;x_{t}\right]\right)=\sigma \left(W_{hz}h_{t-1}+W_{tz}x_{t}\right)\\ r_{t}= & \;\sigma \left(W_{r}\cdot \left[h_{t-1},\;x_{t}\right]\right)=\sigma \left(W_{hr}h_{t-1}+W_{tr}x_{t}\right)\\ {\tilde{h}}_{t}= & \;\tanh\left(W_{h}\cdot \left[r_{t}⊗h_{t-1},\;x_{t}\right]\right)=\tanh\left(W_{hh}\left(r_{t}⊗h_{t-1}\right)+W_{th}x_{t}\right)\\ h_{t}= & \;\left(1-z_{t}\right)⊗h_{t-1}+z_{t}⊗{\tilde{h}}_{t} \end{array}$$

where $r_{t}$ and $z_{t}$ denote the reset gate and the update gate. $W_{z}$, $W_{r}$ and $W_{h}$ are learnable weight matrices and ${\tilde{h}}_{t}$ is the proposed hidden state. σ(.) and tanh(.) correspond to the logistic sigmoid and hyperbolic tangent function, respectively and ⊗ is the symbol for element-wise multiplication.

Both encoder and decoder are RNNs and they are jointly trained to predict the next value of a target sequence given a source sequence. In particular, two loss functions were used to achieve a multitask objective. $\bar{ABPM}$ and $\hat{ABPM}$ points were decomposed by their values ${\bar{ABPM}}^{v}$ and ${\hat{ABPM}}^{v}$, respectively, and classes ${\bar{ABPM}}^{c}$ and ${\hat{ABPM}}^{c}$, respectively.

#### 2.3.2. Model Inputs

**Figure 5:**
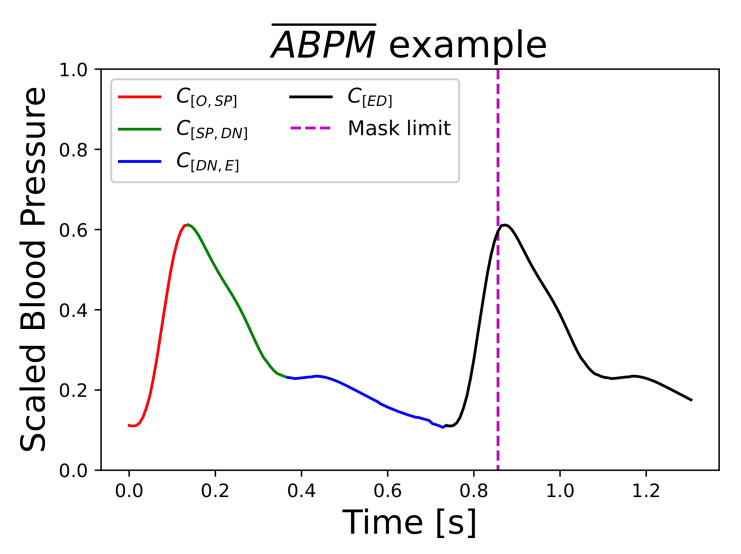
Fixed-size target signal composed by a completed $\bar{ABPM}$ and its repetition. Red, green, blue, and black lines correspond to classes [onset - systolic peak], [systolic peak—dicrotic notch], [dicrotic notch—end] and [Ended], respectively. Vertical magenta dotted line represents the end of the error’s mask.

Model inputs were defined as 5-s random window signals. PPG and PPG were scaled on-the-fly, independently, in the range [0, 1]. Furthermore, ${\bar{ABPM}}^{v}$ was also scaled in [0, 1] but considering global minimum and maximum values in $\bar{ABPM}$ dataset. Because of the different durations of $\bar{ABPM}$, an homogenization step was performed. To the largest $\bar{ABPM}$’s duration 0.12 s (15 time-steps) were added. Thus, the fixed length was set to 1.312 s (164 time-steps). Each $\bar{ABPM}$ was repeated until the fixed length was reached, as shown on Figure 5. To all repeated points it was assigned a new class called [*ended*] ($C_{\left[ED\right]}$), thus increasing the number of classes to 4.

To accelerate the training, as the objective was to predict only one $\hat{ABPM}$, a mask vector with ones and zeros was created. ${\hat{ABPM}}^{v}$ error was masked with it to only penalize the nonrepeated ${\bar{ABPM}}^{v}$ adding 0.12 s (15 time-steps). In Section 2.3.4 this mask will be considered. An example of the limit of the mask is shown in Figure 5 with a vertical magenta dotted line. Nevertheless, ${\hat{ABPM}}^{c}$ was penalized over the whole fixed-size target signal to force the prediction of the $C_{\left[ED\right]}$ class.

#### 2.3.3. Model Architecture

**Figure 6:**
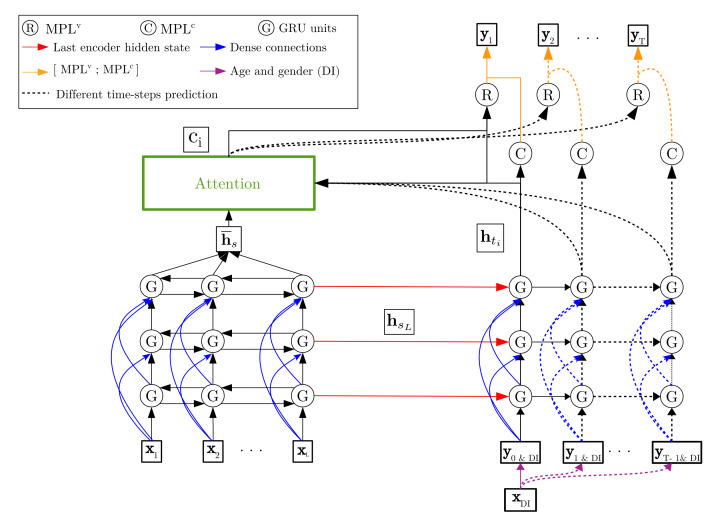
Model architecture.

Figure 6 shows the model architecture. It is constituted by three main parts: encoder, decoder, and attention modules. The encoder consists of three bidirectional GRU (Bi-GRU) layers, while decoder consists of three GRU and two multiperceptron layers (MPL) ($MPL^{v}$ and $MPL^{c}$, respectively). Both encoder and decoder have dense connections [39] to improve the information flow between layers (blue arrows, Figure 6). Input $X_{l}$, with l∈[1,L], is the mentioned 5-s PPG and PPG’ input signal. The whole encoder outputs (${\bar{h}}_{s}$) go to attention module. In addition, the last hidden state ($h_{s_{L}}$) from each encoder GRU layer is used to initialize the hidden states of the corresponding decoder GRU layer (red arrows, Figure 6). The output of last decoder GRU layer ($h_{t_{i}}$) is sent to the both attention module and $MPL^{c}$ layer. Context vector ($c_{i}$) and $h_{t_{i}}$ are concatenated and transferred to $MPL^{v}$. Finally, $MPL^{v}$ and $MPL^{c}$ outputs are concatenated (orange arrows, Figure 6) to produce a prediction time-step ($y_{i}$). Age and gender demographic information vector ($X_{DI}$) is concatenated at each time-step with $y_{i}$ to conform the decoder inputs ($y_{i\&DI}$). In particular, $y_{0}$ is a vector full of ones used to indicate the start of a prediction.

In detail, the attention mechanism used in this work refers to the *Luong Attention* [38], where $c_{i}$ is the weighted sum between an attention weight vector ($a_{i}$) and $h_{t_{i}}$:

(4) $$c_{i}=\sum_{i=0}^{T}a_{i}h_{t_{i}}$$

where $a_{i}$ is computed and normalized using the *softmax* function:

(5) $$a_{i}=\exp\left(score\left(h_{t_{i}},\;h_{s}\right)\right)/\sum_{s^{\prime }\in s}^{}\exp\left(score\left(h_{t_{i}},\;h_{s^{{}^{\prime }}}\right)\right)$$

where $h_{s}$ is each encoder output and $score(h_{t_{i}},\;h_{s})$ is the *general context-based* function:

(6) $$score\left(h_{t_{i}},\;h_{s}\right)=h_{t_{i}}^{Т}\;W\;h_{s}$$

in which *W* is also a weight matrix of a MPL.

#### 2.3.4. Loss Functions

As mentioned before, the model was trained to produce ${\hat{ABPM}}^{v}$ and ${\hat{ABPM}}^{c}$. The difference between ${\hat{ABPM}}^{v}$ and ${\bar{ABPM}}^{v}$ was penalized using mean squared error (MSE) function:

(7) $$MSE=\frac{1}{N}\sum_{j=1}^{N}\frac{1}{M}\sum_{i=1}^{M}{\left({\bar{ABPM}}_{ji}^{v}-{\hat{ABPM}}_{ji}^{v}\right)}^{2}$$

while the difference between ${\hat{ABPM}}^{c}$ and ${\bar{ABPM}}^{c}$ was penalized with the categorical cross-entropy [40] function (CE):

(8) $$CE=\frac{1}{N}\frac{1}{T}\sum_{j=1}^{N}\sum_{i=1}^{T}{\bar{ABPM}}_{ji}^{c}\log\left({\hat{ABPM}}_{ji}^{c}\right)$$

where, for Equations (7) and (8), *N*, *M*, and *T* correspond, respectively, to the number of samples, the mask length previously mentioned and the fixed input length. Finally, the training loss function was defined as:

(9) $$Loss_{train}=MSE+\lambda \;CE$$

in which λ was a constant empirically determined to 0.01.

### 2.4. Hyperparameters and Experimental Settings

The encoder Bi-GRU units per layer are 4, 20 and 100, respectively. Similarly, decoder GRU units per layer are 8, 40, and 200. In addition, $MPL^{v}$ and $MPL^{c}$ layers have 1 and 4 units, respectively, with ELU [41] activation functions. $MPL^{v}$ and $MPL^{c}$ outputs were assigned to ${\hat{ABPM}}^{v}$ and ${\hat{ABPM}}^{c}$, respectively. In particular, $MPL^{v}$ output was previously normalized with a *softmax* function. Adam optimizer [42] was chosen to update the model parameters and the learning rate (LR) value was $10^{-3}$. LR was scaled by 50% after a patience of 25 epochs without improvement in the loss. Training was stopped when patience reaches 50 epochs. The batch size was set to 48.

Weights of $MPL^{v}$, $MPL^{c}$ and Attention layers are initialized from $U(-\sqrt{w},\sqrt{w})$ and weights for GRU layers are initialized from $U(-\sqrt{k},\sqrt{k})$ where

(10) $$w=\frac{1}{\#\;Layer\;input\;size}\;,\;k=\frac{1}{\#\;GRU\;units}$$

In particular, for the weights corresponding for the transition matrix of the GRU layers ($W_{hz},W_{hr},W_{hh}$, from Equation (3)) a random orthogonal initialization scheme was selected [43].

Three scenarios were proposed to evaluate the impact of $X_{DI}$ and the split of segments by subject. For the first and second scenarios, the mixing of segments from the same subject between train and test sets ($Mix_{no}$) was not allowed. For the first scenario neither $X_{DI}$ was provided. Then, for the second scenario $X_{DI}$ information was added. Finally, for the third scenario, it was also allowed that the train and test sets had segments from same subjects ($Mix_{yes}$). The scenarios were named: $Mix_{no}$, $Mix_{no}+DI$ and $Mix_{yes}+DI$, respectively. For scenarios $Mix_{no}$ and $Mix_{no}+DI$ the test set was formed by the segments corresponding to 20% of the subjects. For scenario $Mix_{yes}+DI$ the test set was conformed by 20% of the segments, independently of the subjects. Each scenario was cross-validated 5 times.

### 2.5. Evaluation

Firstly, to evaluate the cuff-less calibration in respect to real ABP, ${\bar{ABPM}}^{v}$ and ${\hat{ABPM}}^{v}$ were restored to the minimum and maximum ABP global scale, then using ${\hat{ABPM}}^{v}$ the DBP, DN and SBP were computed (${\hat{ABPM}}^{\mathrm{DBP}}$, ${\hat{ABPM}}^{\mathrm{DN}}$ and ${\hat{ABPM}}^{\mathrm{SBP}}$, respectively). ${\hat{ABPM}}^{\mathrm{DBP}}$ was computed as the mean between the first and last value of ${\hat{ABPM}}^{v}$. ${\hat{ABPM}}^{\mathrm{DN}}$ and ${\hat{ABPM}}^{\mathrm{SBP}}$ were considered as the last occurrence of the class $C_{\left[SP,DN\right]}$ in ${\hat{ABPM}}^{c}$ and maximum value in ${\hat{ABPM}}^{v}$, respectively. In this sense, the evaluation metrics for ${\hat{ABPM}}^{\mathrm{DBP}}$, ${\hat{ABPM}}^{\mathrm{DN}}$, ${\hat{ABPM}}^{\mathrm{SBP}}$ were root mean squared error (RMSE), mean absolute error (MAE), standard deviation of the errors (STD), and the coefficient of determination ($R^{2}$):

(11) $$RMSE=\sqrt{MSE}=\sqrt{\frac{1}{N}\sum_{i=1}^{N}{\left(z_{i}-{\hat{z}}_{i}\right)}^{2}}$$

(12) $$MAE=\frac{1}{N}\sum_{i=1}^{N}\left|\left(z_{i}-{\hat{z}}_{i}\right)\right|$$

(13) $$R^{2}=1-\left[\sum_{i=1}^{N}\left(z_{i}-{\hat{z}}_{i}\right)/\sum_{i=1}^{N}\left(z_{i}-{\bar{z}}_{i}\right)\right]$$

where ${\bar{z}}_{i}$ is the mean of $z_{i}$ and ${\hat{z}}_{i}$ is the estimated value. Secondly, DN time occurrence ($DN^{\mathrm{TO}}$) and pulse duration were also evaluated with RMSE, MAE, and $R^{2}$ metrics. $DN^{\mathrm{TO}}$ and pulse duration were computed as last and first occurrence of classes $C_{\left[DN,E\right]}$ and $C_{\left[ED\right]}$, respectively, in ${\hat{ABPM}}^{c}$. Finally, $\hat{ABPM}$ pulse values were evaluated with RMSE and MAE, while $\hat{ABPM}$ pulse waveforms were evaluated with the Pearson’s coefficient of correlation (*R*). When $\bar{ABPM}$ and $\hat{ABPM}$ had different durations, the shorter one was considered for the evaluation. *R* is defined as:

(14) $$R=\sum_{i=1}^{T}\left(x_{i}-\bar{x}\right)\left(y_{i}-\bar{y}\right)/\sqrt{\sum_{i=1}^{T}{\left(x_{i}-\bar{x}\right)}^{2}{\left(y_{i}-\bar{y}\right)}^{2}}$$

where *x* and *y* correspond to $\bar{ABPM}$ and $\hat{ABPM}$, $\bar{x}$ and $\bar{y}$ theirs mean, and *T* the considered duration.

## 3. Results

**Figure 7:**
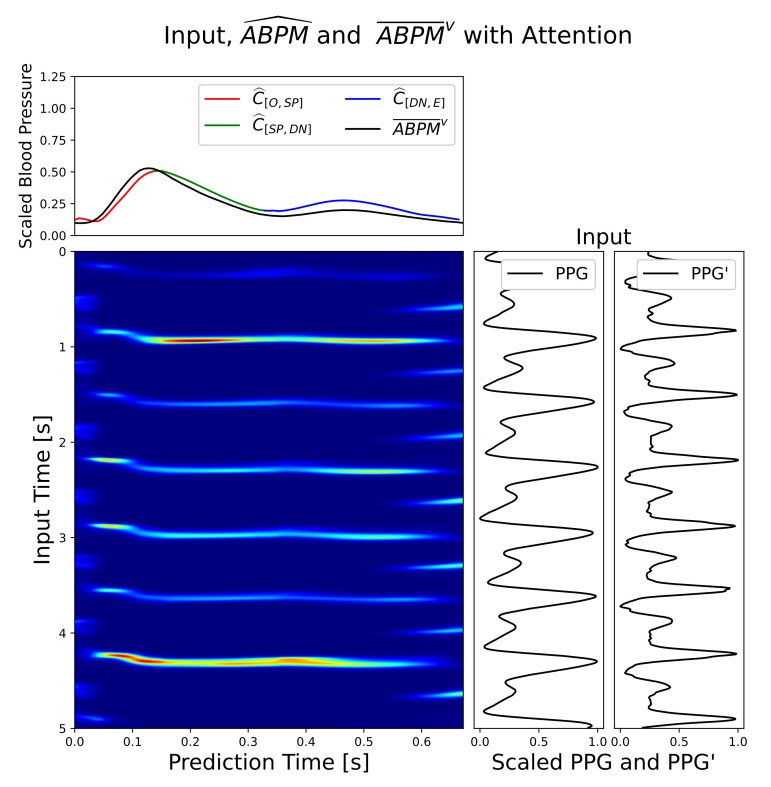
Relationship between photoplethysmogram signals (PPG) and PPG input signals and $\hat{ABPM}$ via attention weight’s heatmap. Additionally, ${\bar{ABPM}}^{v}$ is shown in comparison with $\hat{ABPM}$.

**Figure 8:**
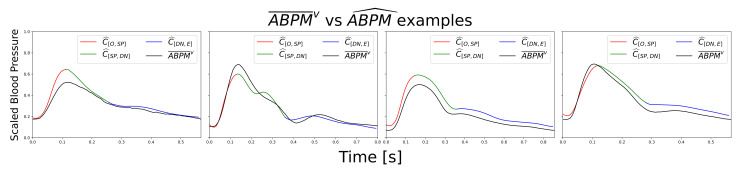
Comparison between different ${\bar{ABPM}}^{v}$ and $\hat{ABPM}$ examples.

Figure 7 shows an input segment, attention weights, and output test example from the $Mix_{no}+DI$ scenario. Upper left plot compares ${\bar{ABPM}}^{v}$ with respect to $\hat{ABPM}$. Red, green, and blue points represent ${\hat{ABPM}}^{c}$, while the black line is ${\bar{ABPM}}^{v}$. In the lower left plot the grade of intensity of heat map points determines the level of attention applied to the input (lower right plot) by the model to produce each $\hat{ABPM}$ point. In addition, Figure 8 shows other test samples with very different morphologies predicted. It is important to corroborate that the model did not learn a global average morphology.

Table 1 shows the obtained values of ${\hat{ABPM}}^{\mathrm{DBP}}$, ${\hat{ABPM}}^{\mathrm{DN}}$, and ${\hat{ABPM}}^{\mathrm{SBP}}$ results. In particular, ${\hat{ABPM}}^{\mathrm{DBP}}$ and ${\hat{ABPM}}^{\mathrm{SBP}}$ assessment refers to a cuff-less calibration task. In ascending order, they were $Mix_{no}$, $Mix_{no}+DI$, and $Mix_{yes}+DI$. Regarding the time occurrences assessment from Table 2, there was not a clear difference between $Mix_{no}$ and $Mix_{no}+DI$ scenarios. Despite the mean of the metrics being slightly better for the $Mix_{no}$ scenario, they also show a larger standard deviation. On the contrary, $Mix_{yes}+DI$ scenario show better results. Respecting the evaluation of waveforms and values of $\hat{ABPM}$ presented in Table 3 there was again a clear improvement in performance for the scenario $Mix_{yes}+DI$, followed by scenarios $Mix_{no}+DI$ and $Mix_{no}$.

Table 4 shows the results regarding the British Hypertension Society (BHS) standards [44] using prediction of each fold per scenario. BHS define thresholds (i.e., 5, 10, and 15 mmHg) to inform the cumulative error percentage and determine the grade of a device when the BP is measured. DBP estimation during $Mix_{yes}+DI$ scenario achieves grade B, requiring 3.4% for the range <5 mmHg to achieve grade A. $Mix_{no}$ and $Mix_{no}+DI$ scenarios achieve grade C, lacking 8.2% and 4.5%, respectively, for the range <5 mmHg to achieve grade B.

**Figure 9:**
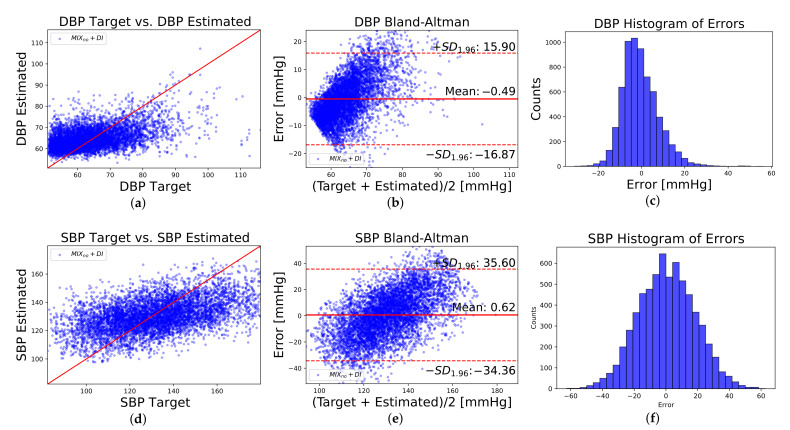
Regression plots (**a**,**d**), Bland–Altman plots (**b**,**e**), and histograms of errors (**c**,**f**), corresponding to $Mix_{no}+DI$ scenario and DBP and SBP values.

Bland–Altman plots were performed using SBP and DBP predictions of each of the 5 fold per scenario. Bland–Altman results are shown in Table 5 in terms of mean (μ) and limits of agreement (μ±1.96σ). In particular, Figure 9 shows regression plots, Bland–Altman plots, and histograms of errors corresponding to $Mix_{no}+DI$ scenario.

## 4. Discussion

In the present work, the $\bar{ABPM}$ was estimated by combining the time-series of the PPG and DI of each subject. Firstly, the $\bar{ABPM}$ signal was computed and paired to its corresponding PPG signal and DI. Secondly, a model with sequence-to-sequence architecture and attention mechanism was proposed to transfer the information from the optical domain to the pressure domain. Results show the capacity of the proposed method to simultaneously estimate both morphology and calibration values of the ABP signal.

To the best of our knowledge, a distinction is made in the literature between calibration methods. Depending on whether or not data from the same subject are used for training and testing, they are called calibration-based (cal-based) or calibration-free (cal-free), respectively. In this sense, hereafter $Mix_{yes}+DI$ and $Mix_{no}+DI$ scenarios refer to cal-based and cal-free, respectively. Table 6 presents a comparison, in calibration terms, with other studies. Nevertheless, because of different evaluation metrics, dataset sizes, and signal sources, the comparison is not easy and direct. Studies that reported lowest errors are those with fewer number of subjects and in which the restriction to use subject data in both training and training sets was not explicit or was not applied. Particularly, in Chan et al. [18], mean error (ME) was used as a metric and the dataset was unspecified. In Kurylyak et al. [20], despite that only PPG signal was used, the dataset consisted only of 15,000 beats and no information about number of subject was given. In Chowdhury et al. [21] the dataset consists of 226 records, with a signal duration of 2.1 s and corresponding to 126 subjects. Methods in Chan et al. [18], Kurylyak et al. [20] and Chowdhury et al. [21] use the feature extraction approach. On the contrary, in Eom et al. [24] a deep learning model with the capacity to take raw multi signal inputs was proposed. However, the dataset was composed of only 15 subjects, without restricting the use of data from the same subjects to train and test.

Works that have used largest amount of subjects were [19,22,23,26] (410, 572, 1000, and 510 subjects, respectively). In Monte-Moreno [22], estimations of SBP and DBP were obtained using only features extracted from the PPG, combined with the age, weight, and body mass index information of the subjects. The author did not make explicit any subject’s data restriction (cal-based scenario). Assessments were reported in terms of $R^{2}$ metric and results reached a grade B under the standards. On the contrary, results from [19,22,26] reported results much more similar with those obtained in the present work and also expressed subject’s data restriction for train and test set. In Ruiz-Rodríguez et al. [23], only a cal-free scenario was reported and errors were informed in terms of a Bland–Altman test. Limits of agreement for SBP and DBP were [−40.91, 34.94 ] and [−20.68, 13.38] mmHg, respectively, and mean values were −2.98 and −3.65 mmHg, respectively. Although they had a lot of clinical information available in their database, this information was not included in their model when estimating BP values. Particularly, in Kachuee et al. [19] were reported the lowest errors in both cal-free and cal-based scenarios. Nevertheless, ECG information was necessary jointly with the PPG time series, followed by a feature extraction step to get estimations. On the contrary, in Slapniča et al. [26], where a leave-one-subject-out experiments were performed, only PPG raw signal was necessary. The dataset used in [26] was nearly the half used in this work, and except for $SBP_{MAE}$ evaluation in the cal-based scenario, the results presented here are better. Additionally, in no case authors of studies [19,22,23,26] reported a limitation on the number of records per subject or the total duration per record. In these terms, we suggest our work is less biased.

Table 7 shows a comparison between different methods and results that were focused on the continuous ABP and our results given in Table 3. In Sideris et al. [27] there were 42 subjects and records analyzed from MIMIC database, and each record was composed of two segments. Furthermore, a completely personalized approach was proposed, in which as many different models as subjects were created. On the contrary, in our approach just a single model needs to be trained. In Sadrawi et al. [28] the proposed DCAE model was trained with 18 subjects from closed data. Additionally, while DCAE model only accepted fixed input length, the methodology proposed in the present work does not have that limitation. Although in Sideris et al. [27] and Sadrawi et al. [28] MAE and RMSE values were lower than those in the present work, the number of subjects evaluated was lower and there was no subject’s data restriction between train and test sets.

It is important to mention that while all PPG signals came from the finger, it is unknown from which specific site of the arterial tree BP signals were recorded. Nevertheless, future studies could improve the results by specifying the sites of the source and target signals. Furthermore, the information about devices and filters used during data collection is also unknown for both PPG and ABP signals. Therefore, in addition to the fact that the type of drug supplied or the existence of previous pathologies is also unknown, the scenario does not meet the standards of a rigorous medical protocol.

Finally, compared to the previous studies found in the literature, the presented architecture allows for the use of both raw signals and DI (age and gender) as inputs. An improvement in results can be observed when DI is considered (Table 1 and Table 3). Furthermore, without any modification in the architecture, other characteristics of the subject could be incorporated, such as ethnicity, weight, or height. Despite that many of them are present in the MIMIC-III CDB, the final number of subjects with extra information and also good quality records was less than 30% of the total used. Pre-existing conditions such as diabetes, chronic kidney disease, smoking, and dyslipidemia also could be incorporated.

## 5. Conclusions

In this paper, a new deep learning architecture to estimate the average arterial blood pressure morphology ($\bar{ABPM}$) is proposed. The proposed methodology, for each point that conforms the $\bar{ABPM}$, estimates the blood pressure value and classifies it according to the stage of the cardiac cycle to which belongs. To the best of our knowledge, this is a contribution to the literature because most of the existing approaches only estimate diastolic and systolic values. The methodology presented here also allows simultaneous use of subject demographic information and raw photoplethysmogram signal from the finger as model input. Further studies are needed with more specific databases in order to expand the presented results. In addition, the source code is shared concerning the reproducibility of the results. Finally, as a potential research direction, this methodology could be adapted to mobile devices where only one source signal is required.

## Figures and Tables

**Table 1:** Mean and standard deviation of the metrics used to evaluate the diastolic (DBP), dicrotic notch (DN), and systolic blood pressure (SBP) errors.

Marker	Scenario	$R^{2}$	RMSE	MAE	STD
DBP	$Mix_{no}$	0.10 ± 0.03	8.88 ± 0.27	7.01 ± 0.23	8.84 ± 0.25
	$Mix_{no}+DI$	0.19 ± 0.04	8.47 ± 0.29	6.57 ± 0.20	8.43 ± 0.29
	$Mix_{yes}+DI$	**0.41** ± 0.04	**7.40** ± 0.20	**5.56** ± 0.18	**7.32** ± 0.17
DN	$Mix_{no}$	0.29 ± 0.02	11.23 ± 0.44	8.72 ± 0.31	11.15 ± 0.38
	$Mix_{no}+DI$	0.32 ± 0.04	10.95 ± 0.27	8.54 ± 0.37	10.84 ± 0.26
	$Mix_{yes}+DI$	**0.50** ± 0.02	**9.67** ± 0.17	**7.08** ± 0.19	**9.63** ± 0.15
SBP	$Mix_{no}$	0.17 ± 0.04	18.20 ± 0.52	14.55 ± 0.56	18.04 ± 0.54
	$Mix_{no}+DI$	0.19 ± 0.05	18.07 ± 0.60	14.39 ± 0.42	17.87 ± 0.40
	$Mix_{yes}+DI$	**0.39** ± 0.05	**15.96** ± 0.60	**12.08** ± 0.36	**15.67** ± 0.50

Root mean squared error (RMSE), mean absolute errors (MAE), and standard deviation of the errors (STD) in mmHg.

**Table 2:** Mean and standard deviation of the metrics used to evaluate the errors in the dicrotic notch time occurrence ($DN^{\mathrm{TO}}$) and the pulse duration from the estimated mean arterial blood pressure pulse morphology ($\hat{ABPM}$).

Scenario	${\mathrm{DN}}^{\mathrm{TO}}$			Pulse Duration		
	$R^{2}$	RMSE	MAE	$R^{2}$	RMSE	MAE
$Mix_{no}$	0.55 ± 0.10	33 ± 3	24 ± 2	0.97 ± 0.02	22 ± 8	15 ± 9
$Mix_{no}+DI$	0.54 ± 0.05	35 ± 3	25 ± 1	0.97 ± 0.01	24 ± 5	16 ± 4
$Mix_{yes}+DI$	**0.61** ± 0.02	33 ± 1	**23** ± 1	**0.98** ± 0.01	**18** ± 2	**11** ± 1

RMSE and MAE in ms.

**Table 3:** Mean and standard deviation of the metrics used to evaluate the waveform and value errors for each estimated arterial blood pressure pulse morphology ($\hat{ABPM}$).

Scenario	$\hat{\mathrm{ABPM}}$		
	R	RMSE	MAE
$Mix_{no}$	0.98 ± 0.002	10.39 ± 0.11	9.06 ± 0.09
$Mix_{no}+DI$	0.98 ± 0.001	10.26 ± 0.11	8.89 ± 0.10
$Mix_{yes}+DI$	0.98 ± 0.001	**8.65** ± 0.20	**7.37** ± 0.21

RMSE and MAE in mmHg.

**Table 4:** Comparison with the British Hypertension Society (BHS) Standard.

Scenario		Cumulative Error Percentage		
		<5mmHg	<10mmHg	<15mmHg
$Mix_{no}$	DBP	41.8%	76.6%	92.9%
	SBP	21.6%	42.0%	59.3%
$Mix_{no}+DI$	DBP	45.5%	80.2%	93.5%
	SBP	21.3%	41.9%	58.6%
$Mix_{yes}+DI$	DBP	56.6%	86.0%	95.5%
	SBP	29.6%	53.2%	70.3%
**BHS** [44]	Grade A	60%	85%	95%
	Grade B	50%	75%	90%
	Grade C	40%	65%	85%

**Table 5:** Limits of agreements(μ±1.96σ) and means (μ) for Bland–Altman plots.

	Scenario	Limits	Mean
DBP	$Mix_{no}$	[−17.80, 16.77]	−0.52
	$Mix_{no}+DI$	[−16.87, 15.90]	−0.49
	$Mix_{yes}+DI$	[−14.23, 14.48]	0.13
SBP	$Mix_{no}$	[−32.85, 37.27]	2.21
	$Mix_{no}+DI$	[−34.36, 35.60]	0.62
	$Mix_{yes}+DI$	[−28.78, 32.69]	1.95

Limits and Mean in mmHg.

**Table 6:** Comparison with related works in term of cuff-less calibration results.

Author	Dataset	Method	Input	Signals	Calibration	Error	
						DBP	SBP
Chan et al. [18]	Unspecified	Linear regression	Feature	ECG	Cal-based	ME: 4.08	ME: 7.49
				PPG		STD: 5.62	STD: 8.82
Kurylyak et al. [20]	MIMIC	Neural network	Feature	PPG	Cal-based	MAE: 2.21	MAE: 3.80
	(15,000 beats)					STD: 2.09	STD: 3.46
Chowdhury et al. [21]	Dataset from [45] (126 subjects)	Gaussian process regression (GPR)	Feature	PPG	Cal-based	MAE: 1.74	MAE: 3.02
						RMSE: 3.59	RMSE: 6.74
						R: 0.96	R: 0.95
Eom et al. [24]	Own data (15 subjects)	Deep learning (CNN+GRU +Attention)	Raw	ECG	Cal-based	MAE: 3.33 RMSE: 3.42	MAE: 4.06 RMSE: 4.04
				BCG			
				PPG			
Monte-Moreno [22]	Own data (410 subjects)	Random Forest (RF)	Feature	PPG	Cal-free	$R^{2}$: 0.89	$R^{2}$: 0.91
Kachuee et al. [19]	MIMIC-II (1000 subjects)	AdaBoost	Feature	ECG PPG	Cal-free	MAE: 5.35	MAE: 11.17
						STD: 6.14	STD: 10.09
						R: 0.48	R: 0.59
					Cal-based	MAE: 4.31	MAE: 8.21
						STD: 3.52	STD: 5.43
						R: 0.57	R: 0.54
Slapničar et al. [26]	MIMIC-III (510 subjects)	Deep learning (ResNet)	Raw	PPG	Cal-free	MAE: 12.38	MAE:15.41
					Cal-based	MAE: 6.88	MAE: 9.43
This work	MIMIC-III Matched Subset (1131 subjects)	Deep learning (Seq2seq +Attention)	Raw	PPG	Cal-free	MAE: 6.57	MAE: 14.39
						STD: 8.43	STD: 17.87
						RMSE: 8.47	RMSE: 18.07
						$R^{2}$: 0.19	$R^{2}$: 0.19
					Cal-based	MAE: 5.56	MAE: 12.08
						STD: 7.32	STD: 15.67
						RMSE: 7.40	RMSE: 15.96
						$R^{2}$: 0.41	$R^{2}$: 0.39

ME, RMSE, STD, and MAE in mmHg.

**Table 7:** Comparison with related works in term of waveform results.

Author	Dataset	Method	Calibration	Error
Sideris et al. [27]	MIMIC (42 subjects)	LSTM	Cal-based	RMSE: 6.0
				STD: 3.26
				R:0.95
Sadrawi et al. [28]	Own data (18 subjects)	DCAE	Cal-based	RMSE: 3.46
				MAE: 2.33
				R: 0.98
This work	MIMIC-III Matched Subset (1131 subjects)	Seq2seq+Attention	Cal-free	RMSE: 10.26
				MAE: 8.89
				R: 0.98
			Cal-based	RMSE: 8.67
				MAE: 7.39
				R: 0.98

RMSE, STD and MAE in mmHg.

## Supplementary Materials

The source codes are available at https://github.com/AguirreNicolas/PPG2IABP and the processed dataset is available at https://doi.org/10.5281/zenodo.4598938.

## Abbreviations

The following abbreviations are used in this manuscript:

ABP	Arterial blood pressure
ABPM	Arterial blood pressure morphology
$\bar{ABPM}$	Average arterial blood pressure pulse morphology
$\hat{ABPM}$	Estimated arterial blood pressure pulse morphology
BP	Blood pressure
BCG	Ballistocardiogram
BHS	British Hypertension Society
CDB	Clinical database
CNN	Convolutional neural network
CVDs	Cardiovascular diseases
DCAE	Deep convolutional auto-encoder
DI	Demographic information
DN	Dicrotic notch
$DN^{\mathrm{TO}}$	Dicrotic notch time occurrence
GPR	Gaussian process regression
GRU	Gated recurrent unit
LSTM	Long-short term memory
LR	Learning rate
MAE	Mean absolute error
NN	Neural network
R	Pearson’s correlation coefficient
RNN	Recurrent neural network
RMSE	Root-mean squared error
$R^{2}$	Coefficient of determination
SQ	Signal quality
STD	Standard deviation of the errors
PAT	Pulse arrival time
PEP	Pre-ejection period
PPG	Photoplethysmography
PPG’	1st derivative of the photoplethysmogram
PPG”	2nd derivative of the photoplethysmogram
PTT	Pulse transit time
PWV	Pulse wave velocity
MWDB	Matched waveform database
